# Intranasal NAP (davunetide) decreases tau hyperphosphorylation and moderately improves behavioral deficits in mice overexpressing α-synuclein

**DOI:** 10.1002/prp2.65

**Published:** 2014-08-06

**Authors:** Iddo Magen, Regina Ostritsky, Franziska Richter, Chunni Zhu, Sheila M Fleming, Vincent Lemesre, Alistair J Stewart, Bruce H Morimoto, Illana Gozes, Marie-Françoise Chesselet

**Affiliations:** 1 Department of Neurology, The David Geffen School of Medicine at UCLA710 Westwood Plaza, Los Angeles, California, 90095-1769; 2 Department of Human Molecular Genetics and Biochemistry, Sackler School of Medicine, Adams Super Center for Brain Studies and Sagol School of Neuroscience, Tel Aviv UniversityTel Aviv, 69978, Israel; 3 Allon Therapeutics Inc.Vancouver, British Columbia, Canada, V6B 2S2; 4 Paladin Labs Inc.100 Blvd Alexis Nihon, Suite 600, St Laurent, Quebec, Canada, H4M 2P2; 5 Celerion621 Rose St, Lincoln, Nebraska, 68502

**Keywords:** Aggregates, buried pellet, challenging beam, microglia, NAP, open field, pole, tau, α-synuclein

## Abstract

Genome-wide association studies have identified strong associations between the risk of developing Parkinson's disease (PD) and polymorphisms in the genes encoding α-synuclein and the microtubule-associated protein tau. However, the contribution of tau and its phosphorylated form (p-tau) to α-synuclein-induced pathology and neuronal dysfunction remains controversial. We have assessed the effects of NAP (davunetide), an eight-amino acid peptide that decreases tau hyperphosphorylation, in mice overexpressing wild-type human α-synuclein (Thy1-aSyn mice), a model that recapitulates aspects of PD. We found that the p-tau/tau level increased in a subcortical tissue block that includes the striatum and brain stem, and in the cerebellum of the Thy1-aSyn mice compared to nontransgenic controls. Intermittent intranasal NAP administration at 2 *μ*g/mouse per day, 5 days a week, for 24 weeks, starting at 4 weeks of age, significantly decreased the ratio of p-tau/tau levels in the subcortical region while a higher dose of 15 *μ*g/mouse per day induced a decrease in p-tau/tau levels in the cerebellum. Both NAP doses reduced hyperactivity, improved habituation to a novel environment, and reduced olfactory deficits in the Thy1-aSyn mice, but neither dose improved the severe deficits of motor coordination observed on the challenging beam and pole, contrasting with previous data obtained with continuous daily administration of the drug. The data reveal novel effects of NAP on brain p-tau/tau and behavioral outcomes in this model of synucleinopathy and suggest that sustained exposure to NAP may be necessary for maximal benefits.

## Introduction

Evidence points toward a possible interplay between the microtubule-associated protein tau (MAPT) and α-synuclein in the pathology of sporadic Parkinson's disease (PD). Genome-wide association studies have identified a high association between variations in MAPT and PD, with α-synuclein being the major risk gene for PD, and MAPT the second risk gene (Simon-Sanchez et al. 2009). Tau was hyperphosphorylated in striata from PD patients (Wills et al. 2010) and in several PD mouse models overexpressing α-synuclein (Haggerty et al. 2011; Kaul et al. 2011; Wills et al. 2011). Even though a genetic lack of tau did not improve motor deficits in mice overexpressing α-synuclein under the Thy-1 promoter (Thy1-aSyn mice) (Morris et al. 2011), in a cellular model of synucleinopathy, tau enhanced aggregation and toxicity of α-synuclein (Badiola et al. 2011). Thus, compounds regulating tau hyperphosphorylation may be beneficial in PD.

We have previously observed (Fleming et al. 2011) beneficial effects in the Thy1-aSyn mouse model overexpressing α-syuclein of intranasal administration of NAP (NAPVSIPQ; davunetide or AL-108), a neuroprotective peptide derived from activity-dependent neuroprotective protein (ADNP; Bassan et al. 1999) which stabilizes microtubules and decreases tau hyperphosphorylation (Matsuoka et al. 2007, 2008; Vulih-Shultzman et al. 2007; Shiryaev et al. 2009; Jouroukhin et al. 2012, 2013). In our previous pilot study, 2 *μ*g/mouse NAP given daily for 8 weeks to Thy1-aSyn mice improved motor coordination deficits and α-synuclein pathology in the substantia nigra (SN) (Fleming et al. 2011).

In other models, NAP showed brain bioavailability and stability (Gozes et al. 2000, 2005; Morimoto et al. 2009) after intranasal administration, and provided neuronal (Zemlyak et al. 2009) and glial protection (Idan-Feldman et al. 2011). In addition to reducing tau hyperphosphorylation, NAP modulates microtubule stability through interaction with neuronal and glial tubulin (Divinski et al. 2004, 2006; Gozes and Divinski 2004, 2007), while enhancing tau–microtubule interaction (Oz et al. 2012; Quraishe et al. 2013) and protecting axonal transport (Jouroukhin et al. 2013; Quraishe et al. 2013). Inhibition of amyloid *β*-induced tau hyperphosphorylation was also reported (Shiryaev et al. 2011). Importantly, NAP is well tolerated in humans and has been administered to patients with progressive supranuclear palsy without major side effects (Morimoto et al. 2013; Boxer et al. 2014). Taken together, these data warranted further exploration of the effects of NAP on tau phosphorylation and behavioral deficits in the Thy1-aSyn mice.

In the present study, we used a longer duration of intranasal administration (24 weeks) of both the original lower dose of 2 *μ*g/mouse per day and a higher dose of 15 *μ*g/mouse per day, as well as vehicle, in Thy1-aSyn mice and their wild-type (WT) littermates. To accommodate the longer treatment duration and in view of beneficial effects with this regimen in previous studies (cited above), the drug or vehicle were administered 5 days a week (Monday–Friday). Mice were examined for motor deficits at the same time (8 weeks treatment) as in our previous study as well as at the end of treatment. In addition, we examined activity in the open field, nonmotor (olfactory) deficits, and α-synuclein pathology at the end of the longer treatment and also investigated for the first time whether NAP administration could reverse tau hyperphosphorylation in this model and whether it interfered with microglial activation.

## Materials and Methods

### Mice

Animal care was in accordance with the United States Public Health Service Guide for the Care and Use of Laboratory Animals, and procedures were approved by the Institutional Animal Care and Use Committee at the University of California Los Angeles (UCLA). Transgenic mice overexpressing human WT α-synuclein under the Thy-1 promoter (Thy1-aSyn) were developed and crossed into a hybrid C57BL/6-DBA/2 background as described before (Rockenstein et al. 2002). Animals were maintained on the hybrid C57BL/6-DBA/2 background by mating N7 female hemizygous for the transgene with male WT mice on the hybrid background obtained from Charles River Laboratories, Inc. (Wilmington, MA; Fleming et al. 2004, 2006; Fernagut et al. 2007). Male and female mice from the same litters were never bred together. Only male mice were used to avoid inconsistencies due to random inactivation of the X chromosomes (in which the transgene is inserted) in females. Male mice from 30 litters were included in the study and litter sizes ranged from 1 to 8 mice. Mice were enrolled in the study as they were born and genotyped to ensure distribution of offsprings from the same litters among the various experimental groups. Within each litter, mice were randomly assigned to the different drug treatment groups. The different drug treatment groups were coded by an investigator not involved in data analysis and investigators who gathered and analyzed the data were unaware of genotype and treatment throughout the study.

The genotype of all Thy1-aSyn and WT mice was confirmed by polymerase chain reaction (PCR) analysis of tail DNA, at the end of the experiment, with primers for a house keeping gene and human α-synuclein. Animals were maintained on a reverse light/dark cycle with lights off at 10 am and all testing was performed between 12 and 4 pm during the dark cycle under dim light. Food and water were available ad libitum except before and during buried pellet testing.

### Intranasal administration of NAP

**Scheme 1:**
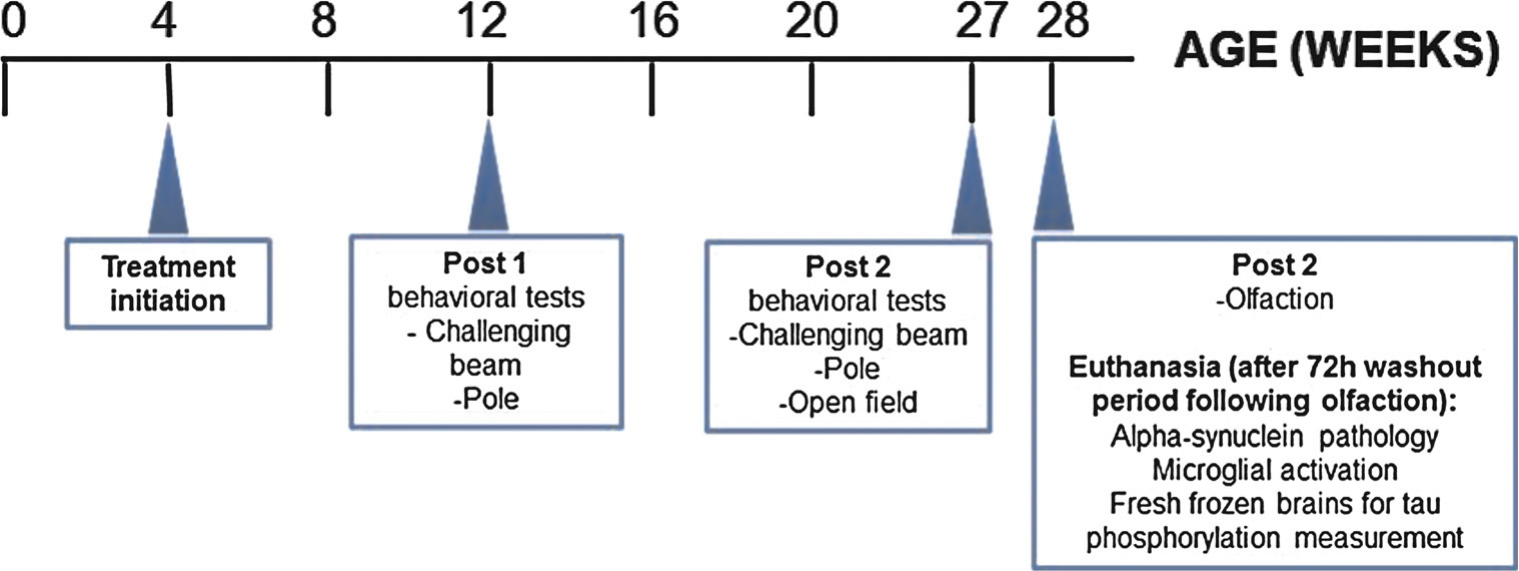
Scheme Summary of study design.

NAP (davunetide) was obtained from Allon Therapeutics Inc., Vancouver, Canada and was dissolved in a vehicle made of sterile water containing 129 mmol/L sodium chloride (7.5 mg/mL), 8 mmol/L citric acid monohydrate (1.7 mg/mL), 17 mmol/L disodium phosphate dihydrate (3.0 mg/mL), and 0.01% benzalkonium chloride (0.2 mg/mL of a 50% solution) (Alcalay et al. 2004) to a final concentration of NAP (0.4 mg/mL). Sodium hydroxide was used to adjust the pH of the dose formulation, to pH 5.0 (±0.5) after addition of NAP. Intranasal NAP administration 2 *μ*g/mouse per day followed a previously published protocol (Fleming et al. 2011). As the 2 *μ*g dose administered daily for 8 weeks was not shown to have an effect on some of the endpoint measurements in the previously conducted pilot study (Fleming et al. 2011), a 15 *μ*g dose, which is in the efficacy range of 0.5–30 *μ*g (Gozes et al. 2000, 2005; Matsuoka et al. 2007, 2008) was also used, coupled with a longer administration period. Mice (4-week-old) were administered 2 or 15 *μ*g per day in a volume of 5 *μ*L (2.5 *μ*L/nare/day) 5 days a week, Monday–Friday (as opposed to the daily administration schedule followed in the pilot study), for 24 weeks. Similar to our previous study, for intranasal administration, each mouse was grasped in a vertical position and the solution was applied using a pipette and 10 *μ*L tip (Denville Scientific, Metuchen, NJ). One droplet was released at the exterior naris and then inhaled by the mouse, and following a short break the drug was administered into the other naris. This method has been extensively validated as producing reliable brain levels of the drug by investigators in the study (Gozes et al. 2000, 2005; Morimoto et al. 2009; Fleming et al. 2011). Investigators who administered the drug were not aware of the nature of the solution (drug, dose, or vehicle). Please see Scheme 1 for summary of study design.

Six groups of male mice were compared, with initial group size in parentheses: WT/vehicle (*n* = 22); Thy1-aSyn/vehicle (*n* = 19); WT/2 *μ*g NAP (*n* = 21); Thy1-aSyn/2 *μ*g NAP (*n* = 20); WT/15 *μ*g NAP (*n* = 17); Thy1-aSyn/15 *μ*g NAP (*n* = 25). The initial group size was determined based on the maximal number of mice needed to detect a 50% drug effect with 80% power and *P* < 0.05, as found by power analysis for each individual test previously performed in Thy1-aSyn mice (see below in the description of the methods); for this exploratory study, our goal was to include a range of tests without preconceived notion of what type of behavior would be most responsive to treatment. Therefore, no correction for multiple tests was included in the power analysis calculation as this would have resulted in prohibitively large number of animals. During the course of the study, sizes of all groups except for WT/vehicle, decreased due to mortality, to the following sizes: Thy1-aSyn/vehicle (*n* = 16); WT/2 *μ*g NAP (*n* = 20); Thy1-aSyn/2 *μ*g NAP (*n* = 16); WT/15 *μ*g NAP (*n* = 16); Thy1-aSyn/15 *μ*g NAP (*n* = 21). Mortality was not related to drug treatment or genotype as it was not different between groups (Fisher's exact test: *P* > 0.05, NS) and in most cases was associated with neck tumors or tooth abscesses. The general condition of the mice was monitored daily during the 24 weeks of intranasal administrations. Weights were measured daily and body temperature was measured weekly during drug administration.

### Behavioral tests

All behavioral testing was performed and rated by investigators unaware of genotype and treatment. Behavior was always measured during the ON treatment period.

#### Open field

The open field test was shown to have high power to detect drug effect; at 7 months of age, less than 10 mice are needed to detect a 50% reduction in distance travelled, time spent in motion and movement velocity, with 80% power (Lam et al. 2011).

Spontaneous activity in an open field (25.5 × 25.5 cm) was monitored for 15 min using an automated system (Truscan system for mice; Coulbourn Instruments, Allentown, PA). Testing took place within the first 2–4 h of the dark cycle after animals had first been habituated to the testing room for 15 min. This was done at 27 weeks of age. The open field was illuminated with anglepoise lamp equipped with a 25-W red bulb. Time spent in motion and number of move episodes were automatically collected by the TruScan software for 3 × 5 min time bins. Time spent in motion per move episode was computed by dividing the total time spent in motion by the number of move episodes. Data were analyzed for both the entire 15-min session and for each one of the 5-min time blocks (Hickey et al. 2005). To confirm that differences in activity levels were not related to differences in body weight, the mice were weighed just before being tested in the open field. Fecal pellets were collected and counted at the end of the session for each mouse, as Thy1-aSyn mice are known to have abnormal gastrointestinal response to novel environment (Wang et al. 2008, 2012).

#### Olfaction test

The buried pellet test was performed as described previously (Fleming et al. 2008) with the exception that mice were tested only for 1 day to avoid compensatory strategies developed over repeated trials by the transgenic mice. Although the Thy1-aSyn mice showed deficits in several olfactory function tests (Fleming et al. 2008), the buried pellet test has the highest power to identify drug effects of all tests detecting deficiencies in the Thy1-aSyn mice – 20 subjects are needed to detect a 50% improvement with 80% power in trial 1. The olfactory testing was performed 5 days after completion of all motor tests (beam and open field) at 28 weeks of age and was completed 3 days prior to sacrifice. Briefly, mice were food restricted and maintained at ∼90% body weight 5 days prior to and during testing. Food restricted mice were given 3–4 g of mouse chow per animal per day depending on weight (monitored each day during food restriction). The surface pellet control test was performed 2 days after the buried pellet test to control for any confounding effect of motor disability or lack of interest in the food pellet. For the buried pellet test, mice were first habituated in the dark to the behavior room in their home cage, with the feeding rack and water bottle removed. A clean mouse cage (15 × 25 × 13 cm) was filled with 3 cm of clean bedding. One piece of sweetened cereal (∼250 mg; Cap'n Crunch®, Quaker, Chicago, IL) was buried in the front left corner of the cage approximately 0.5 cm below the bedding so that it was not visible. A mouse was then placed in the center of the cage and the latency to dig up and begin eating the cereal was measured using a stopwatch. After the trial, each animal was returned back to its home cage. If a mouse did not locate the food pellet within 5 min, the animal was removed, returned to its home cage, and given a score of 5 min. The bedding was changed between mice, and gloves were replaced before touching a fresh bedding to avoid possible contamination of the new bedding with traces of mouse smell on old gloves. Extra care was taken to ensure that food pellets were fresh and could be smelled by the WT mice by replacing them every week, and to ensure that the pellet was buried at approximately the same height below the bedding surface using a ruler. The surface pellet test was set up in a similar way to the buried pellet test except that the piece of cereal was placed on top of the bedding. Number of diggings not in proximity to the pellet, which reflect search strategy not relying on the sense of smell, was also counted, as well as the percentage of mice in each group that detected the pellet within the 5 min cutoff time.

#### Challenging beam

The challenging beam traversal test was previously developed in the UCLA laboratory (Fleming et al. 2004), and is reliably altered in young Thy1-aSyn mice (Fleming et al. 2004, 2006); nine and six subjects are needed to detect a 50% effect on the errors per step averaged across five trials, at 3 months and 6.5 months, respectively. Briefly, the beam consists of four sections (25 cm each, 1 m total length) with different widths. The beam starts at a width of 3.5 cm and gradually narrows to 0.5 cm by 1 cm decrements. Animals were trained to traverse the length of the beam starting at the widest section and ending at the narrowest section, which was leading to the home cage. Beams were cleaned with a disinfectant between mice from different cages. Animals received 2 days of training, prior to testing; on the day of the test a mesh grid (one cm squares) of corresponding width was placed approximately 1 cm over the beam surface. Animals were then videotaped while traversing the grid-surfaced beam and an experimenter blind to genotype and drug condition rated videotapes on slow motion. A different investigator reanalyzed representative videos to verify accuracy. The number of errors per step was measured for each of the five trials on the grid-surfaced beam for each mouse, and was then averaged across five trials. In addition, the number of steps and time to traverse the beam, although not reliably altered in young Thy1-aSyn mice, were measured to ensure that the drug did not have any undesirable effect on motor performance. The beam test was performed 8 and 23 weeks after initiation of treatment. In the second time point, no training was performed as the mice were already familiar with the test.

#### Pole test

The pole test was included in our study because it is robustly altered in young Thy1-aSyn mice; power analysis based on recent data in the UCLA laboratory indicated that 16 mice are necessary to detect a 30% effect on time to turn or descend with 80% power, *P* < 0.05, as an independent measure. Briefly, animals were placed head upward on top of a vertical wooden pole 50 cm long (1 cm in diameter). The base of the pole was made of Styrofoam and was placed in the home cage. When placed on the pole, animals orient themselves downward and descend the length of the pole back into their home cage. Thy1-aSyn and WT mice received 2 days of training that consisted of five trials for each session on time point 1 (8 weeks post treatment, 12 weeks of age). On the test day, animals received five trials; time to orient downward (*t*-turn) and total time to descend (*t*-total) were measured for each trial. The wooden pole as well as the Styrofoam base was cleaned between mice from different cages. In time point 2 (23 weeks post treatment, 27 weeks of age) mice were only tested without further training. If a mouse fell off, slid down, or could not complete the task it was given a default score of 30 sec for time to turn and 60 sec to complete the task (*t*-total). The means of the five trials and each one of the five trials were used for analyses, and the parameters analyzed were time to turn in the pole, time to descend in the pole after turning (calculated as: *t*-total − *t*-turn), and percentage of performers – mice that performed the task within the cutoff of 30 sec for turning and 30 sec for descending after turning.

### Biochemical measurements

#### Protein extraction from brain tissue

Mice were sacrificed 72 h after the last day of treatment; during these 72 h, the vehicle or drug were allowed a washout period. A subset of seven animals from each experimental group was preselected at enrollment to be equally distributed among litters and time of birth in order to avoid any bias. These mice were sacrificed by cervical dislocation followed by decapitation. Brains were removed and the cortex, hippocampus, cerebellum and subcortical region containing the striatum, nucleus accumbens, nucleus basalis, SN, and amygdala were dissected out and snap frozen in liquid nitrogen for further analysis.

Brain samples were homogenized in a 10-fold volume of Ripa buffer (50 mmol/L Tris–HCl buffer, pH 7.4, consisting of 1% Nonidet P40, 150 mmol/L NaCl, 1 mmol/L ethylenediaminetetraacetic acid (EDTA), 0.25% sodium deoxycholate, 1 mmol/L NaF, 1 mmol/L Na_3_VO_4_, 1 mmol/L phenylmethanesulfonylfluoride (PMSF), and protease inhibitor cocktail [crude brain homogenate]). The homogenate was centrifuged at 20,000*g* for 20 min at 4°C, and the supernatant was used as a crude tau fraction (total tau). Initial studies included preparation of soluble and insoluble tau fractions (Matsuoka et al. 2007, 2008), but the paucity of sample material (limited protein content in selected brain regions) precluded conclusive results, hence, analysis concentrated on crude brain homogenates.

Protein concentration was measured by the BCA-200 protein assay kit (Pierce, Rockford, IL).

#### Western blot analyses

Experiments were carried out as stated earlier (Jouroukhin et al. 2012, 2013). In short, sample buffer 5× (125 mmol/L Tris-HCl pH 6.8, 25% *β*-mercaptoethanol [Sigma], 43.5% glycerol, 10% SDS, 0.05% bromophenol blue) was added to protein samples that were further denatured by boiling at 100°C for 5 min. Protein concentrations were adjusted to 1.5 *μ*g/*μ*L (loading was a ∼20 *μ*L per well). Proteins (seven independent samples per experimental group, each representing one animal) were separated by electrophoresis on a 12% (w/v) polyacrylamide gel (Bio-Rad, Hercules, CA) containing 0.1% SDS. Molecular weights were determined using Precision Plus Protein Standards (10–250 kD, Dual Color, Bio-Rad). Each gel included a comparison between two experimental groups (seven samples each) and protein markers (one sample). Following electrophoresis, proteins were transferred to nitrocellulose membrane (Whatman Plc., Kent, UK, Tamar, Israel). Transfer buffer which consisted of (for 1 L) 100 mL Tris-glycine, 700 mL H_2_O, and 200 mL methanol was used. Nonspecific antigen sites were blocked using a solution containing 5% BSA (w/v, Sigma, Rehovot, Israel) in TBST (10 mmol/L Tris pH 8, 150 mmol/L NaCl, and 0.05% Tween 20) for 1 h at room temperature.

The primary antibody was added to the membrane in 3% BSA in TBST and then incubated (4°C for 16 h) with shaking. Primary antibodies that were used included p-Tau^262^ antibody (cat# sc-101813; Santa Cruz Biotechnology, Santa Cruz, CA) 0.5 *μ*g/mL and total tau antibody (Tau5, MBL International, Woburn, MA, cat# AT-5004) 0.5 *μ*g/mL which recognizes all forms of tau (phosphorylated and nonphosphorylated), and the membrane was washed three times in TBST for 5 min. The membrane was then incubated with horseradish peroxidase (HRP)-conjugated secondary antibodies – 1/15,000 polyclonal anti-rabbit or 1/5,000 monoclonal anti-mouse, diluted with 5% milk powder in TBST at room temperature for an hour with shaking. Enhanced chemiluminescence (ECL) solution (Pierce, Rockford, IL) was added for 4 min followed by an exposure onto a hyperfilm (Kodak, Chalon-sur-Saône, France).

Stripping solution (Sigma) was added to the membrane for 5–15 min. This procedure was conducted in order to remove the ECL and antibodies to quantify the actin antibody 1/10,000 dilution (cat# ab170325; Abcam, Cambridge, MA) reaction.

Densitometry used Tina 2.10 g Software (Ray Test, Straubenhardt, Germany). The level of the phosphorylated tau was normalized to total tau and is presented as normalized signal (e.g., p-tau/total tau, with measurements of the 75 kD phosphorylated tau band/total tau at 50 kD). Total tau was normalized to actin. The data were further normalized to the appropriate WT control (or other control as indicated – with control = 100%) for graphic representation.

### Immunohistochemistry

#### α-Synuclein immunohistochemistry

Immunohistochemistry of proteinase K-resistant α-synuclein aggregates was performed at 28 weeks of age (i.e., after 24 weeks of drug treatment) only on Thy1-aSyn mice because WT mice do not display aggregates. Power analysis revealed that at this age, eight mice are needed to detect a 50% drug effect (increase or decrease) on the number of aggregates per 100 *μ*m^2^. Therefore, a subset of 10 mice from each group (distinct from, but, preselected similarly to the ones used for tau phosphorylation measurement) were used for immunohistochemistry. As mentioned earlier, mice were euthanized 72 h after the cessation of treatment. Mice designated for immunohistochemical analyses were deeply anesthetized with pentobarbital (100 mg/kg, i.p.) and intracardially perfused with 0.1 mol/L phosphate-buffered saline (PBS) at room temperature followed by ice cold 4% paraformaldehyde. Brains were rapidly removed, postfixed overnight in 4% paraformaldehyde, cryoprotected in 30% sucrose in 0.1 mol/L PBS until they sank into the bottom of the tube, frozen on powdered dry ice, and stored at −80°C. Free-floating coronal sections (40-*μ*m thick) cut in Leica CM 1800 cryostat (Deerfield, IL) were collected for analysis. For assessment of insoluble α-synuclein aggregates, two sections from the SN and four sections from the olfactory bulb of Thy1-aSyn mice only were washed in 0.1 mol/L PBS, incubated at room temperature for 10 min in 0.1 mol/L PBS containing 5 *μ*g/mL of Proteinase K (Invitrogen, Carlsbad, CA) and then washed with 0.1 mol/L PBS. An alternate set of sections did not receive proteinase K treatment and were only washed in 0.1 mol/L PBS to assess overall staining for α-synuclein. All sections were incubated for 1 h in a blocking solution containing 0.1 mol/L PBS and “mouse on mouse” blocking solution (Vector Laboratories, Burlingame, CA). Sections were then incubated overnight with a primary antibody that recognizes both mouse and human α-synuclein (1 *μ*g/mL mouse anti-α-synuclein, BD Biosciences, San Jose, CA), at 4°C in the presence of 2% normal goat serum. Sections were washed in 0.1 mol/L PBS followed by a 2-h incubation period with a biotinylated secondary antibody, goat anti-mouse IgG (1:200, Vector Laboratories) at room temperature in the presence of 2% normal goat serum. Sections were rinsed in 0.1 mol/L PBS and subsequently incubated in avidin–biotin complex (ABC; Vector Laboratories) for 45 min and rinsed again in 0.1 mol/L PBS followed by an incubation in 0.1 mol/L TB containing 3-3′diamino benzidine (DAB; Sigma, St. Louis, MO) and 0.3% H_2_O_2_ (Sigma) to reveal staining. Sections were rinsed with 0.1 mol/L PBS, mounted on gelatin-coated slides, dehydrated, cleared with xylene, and mounted with Eukitt mounting medium (Calibrated Instruments, Hawthorne, NY).

#### Quantification of α-synuclein positive aggregates in the SN

All quantitative analyses were performed by an investigator unaware of genotype or treatment. In-depth, quantitative analysis of surface area occupied by proteinase K-resistant α-synuclein aggregates was performed for the SN. Acquisition of images in the SN was done at 40× using Stereo Investigator. Two sections from the SN stained for aSyn with proteinase K treatment were used for quantification of aggregates in transgenic mice. The contour of the SN was delineated with a 5× objective using the Stereo Investigator software (MicroBrightField, Colchester, VT) coupled to a Leica DM-LB microscope with a Ludl XYZ motorized stage and *z*-axis microcator (MT12, Heidenheim, Traunreut, Germany). The contour was then divided into four subregions, dorsolateral (DL), dorsomedial (DM), ventrolateral (VL), and ventromedial (VM) as shown in Figure 5A. One image from each subregion in each animal was acquired using Stereo Investigator and a 40× objective used to adjust the focus manually prior to the acquisition of each image. Images of the SN from both hemispheres were transformed to 8 bit files using ImageJ software (ImageJ software, version 1.38x, National Institutes of Health, Bethesda, MD). In order to perform the particle analysis in ImageJ the threshold was set manually to ensure the inclusion of all aggregates. The diameters of aggregates ranged from 0.8 to 30 *μ*m; all sizes were included in the analysis. Inclusions were defined by circularity to avoid inclusion of dust or other artifacts. The area occupied by aggregates in the set threshold was measured using ImageJ.

#### Qualitative assessment of α-synuclein positive aggregates in the olfactory bulb

Proteinase K-resistant α-synuclein aggregates in the glomerular layer of olfactory bulb of Thy1-aSyn mice were assessed qualitatively at 20× magnification using a grading scale from 0 to 2 (0 = no staining, 1 = moderate staining, 2 = intense staining). Only mice with anatomically well-preserved olfactory bulbs were included, and analysis was done only on 2–3 sections out of the four initially used for the staining, because olfactory bulb sections are very fragile after proteinase K treatment. All rating was performed by an investigator familiar with olfactory bulb anatomy who remained unaware of genotype and drug status of the mice during the analysis.

#### IBA-1 immunohistochemistry

For morphological assessment of microglial activation, sections of striatum and SN from PFA-perfused mice (the same ones used for α-synuclein aggregate quantification) were stained for ionized calcium-binding adaptor molecule 1 (IBA-1). Sections were washed in 0.1 mol/L PBS, incubated in 0.5% H_2_O_2_ in methanol for 30 min to inhibit endogenous peroxidase activity, and washed in PBS. Sections were incubated for 1 h in a blocking solution containing 0.1 mol/L PBS, 10% normal goat serum, and 0.5% Triton-X. Sections were then incubated overnight with a primary antibody against IBA-1 (polyclonal rabbit anti-IBA-1; 1:500 dilution; Wako Pure Chemical Industries Ltd., Japan) at 4°C in the presence of 5% normal goat serum. Sections were washed in 0.1 mol/L PBS followed by a 2-h incubation with a biotinylated secondary antibody for IBA-1, goat anti-rabbit IgG (1:200 dilution; Vector Laboratories, Inc.) at room temperature in the presence of 5% normal goat serum. Sections were washed in 0.1 mol/L PBS and subsequently incubated in avidin–biotin complex (ABC; Vector Laboratories) for 45 min and washed again in 0.1 mol/L PBS followed by an incubation in 0.05 mol/L Tris-buffered saline (TBS) containing 3-3′diamino benzidine (DAB; Sigma, St. Louis, MO) and 0.3% H_2_O_2_ (Sigma) to reveal staining. Sections were washed with 0.1 mol/L PBS, mounted on charged glass slides, dehydrated, cleared with xylene, and mounted with Eukitt mounting medium (Calibrated Instruments, Hawthorne, NY).

#### Microglial activation quantification

Microglial activation was quantified in a blinded fashion in two sections of SN or striatum (*N* = 8–11 mice/genotype) by immunostaining with IBA-1 and analysis with StereoInvestigator software (MicroBrightField, Colchester, VT). A Leica DM-LB microscope with a Ludl XYZ motorized stage and *z*-axis microcator (MT12, Heidenheim, Traunreut, Germany) was used for image acquisition. Microglial activation was quantified by measuring cell body diameter. This method is based on evidence that morphologically distinct classes of IBA-1-positive cells could be distinguished based on cell body diameter. In the SN, resting ramified microglial cells had mean cell body diameters of 1–4 *μ*m, hyper-ramified microglia/partially activated microglia had mean cell body diameters of 5–7 *μ*m, and fully activated amoeboid microglial cells had mean cell body diameters of 8–13 *μ*m. Since this technique only focuses on cell body diameter, intermediate stages between hyper-ramified microglia, which have enlarged cell bodies with many branching processes, and partially activated microglia with enlarged cell bodies that have not quite reached the fully activated diameter size cannot be clearly distinguished and the comparison focuses on changes in resting and fully activated cells. A contour was used to delineate the SN under the 5× objective lens to ensure anatomical accuracy. Following delineation, the diameters of microglial cell bodies were measured in the first counting frame and then in every fifth counting frame at 40× magnification. For assessment of microglial activation we used a bootstrapping method similar to a Kolmogorov–Smirnov test using custom MATLAB functions (Efron and Tibshirani 1991). We first determined whether the microglial diameters of transgenic mice were different from WT mice. The bootstrapping uses computational power to get a numerical estimate of the mean distribution and confidence intervals of microglial diameters in a WT mouse. The bootstrap algorithm depends on the notion of a bootstrap sample, which in this case is the group mean microglial diameters of the vehicle-treated WT mice. From this sample we set up a test statistic, M as above. Then, we compared the mean microglial diameters for the transgenic data to the test statistic M. If the microglial diameter of the vehicle-treated Thy1-aSyn mice exceeded 95 of 100 of the randomized diameters, the diameter was considered significantly different from vehicle-treated WT at the *P* < 0.05 level, independent of assumptions about probability distributions. We carried out the same procedure for all experimental groups.

### Statistics

All analyses were conducted with SigmaPlot software (Chicago, IL) for Windows. The level of significance was set at *P* < 0.05. Outliers were removed by performing Grubb's test for outliers, and Fisher's exact test was performed to confirm that the groups did not differ in the percentage of mice excluded. One-way or two-way analysis of variance (ANOVA) with genotype and treatment as factors was used for comparing data not originating from the same animals, while repeated measure (RM) ANOVA was used when test included repeated sampling from the same animals, for example, in the beam. Two-way ANOVA was followed by one-tailed post hoc Dunnett's test (because of unidirectional hypothesis that the drug reverses Thy1-aSyn values to WT values). RM ANOVA was followed by one-tailed Dunnett's test for between subject comparisons and by paired *t*-test for within subject comparisons. When data were not normally distributed (i.e., in tests that include a cutoff value such as pole and buried pellet) nonparametric test (Mann–Whitney *U*) was used, with Bonferroni correction for multiple comparisons. Fisher's exact test with Bonferroni correction for multiple comparisons was used to compare frequencies among groups. Table 1 details the analyses performed for the different data.

**Table 1:** The statistical analyses performed in this study

Data	Test performed
Weight and temperature, progression of deficits in challenging beam	Mixed design ANOVA, genotype, and treatment as between subject factors and age/time point as within subject factor, followed by Dunnett's test
Total tau/p-tau levels, challenging beam (averaged for five trials), open field (total 15-min session), density of IBA-1-positive cells	2 × 3 randomized design ANOVA, genotype, and treatment as fixed factors, followed by Dunnett's test
Challenging beam (individual trials), open field habituation (measured by comparing activity in separate 5-min blocks)	Repeated measures ANOVA (trial × treatment or block × treatment) for each genotype separately, followed by Dunnett's test and paired *t*-test
Surface area of aggregates in subregions of substantia nigra	3 × 4 randomized design ANOVA, subregion, and treatment as fixed factors, followed by Dunnett's test
Latency to find buried pellet, time to turn/descend in pole, qualitative score of aggregates in olfactory bulb	Mann–Whitney *U*-test with Bonferroni correction for multiple comparisons
% of performers in olfaction test and in pole test	Fisher's exact test with Bonferroni correction for multiple comparisons
Distribution analysis of microglial cell diameter	Bootstrapping

For each data analyzed, the statistical test used is specified.

## Results

### NAP does not affect body weight and temperature

Table 2 below summarizes the weights recorded during the 24 weeks of treatment. Mixed design 2 × 3 × 6 ANOVA with genotype and treatment as between subjects factor and age as within subject factor, revealed a main effect of genotype (*F*_1,118_ = 36.57, *P* < 0.001), age (*F*_5,590_ = 37.7, *P* < 0.001), and interaction effect (*F*_10,590_ = 2.09, *P* = 0.02), but not a main effect of treatment (*F*_2,118_ = 0.25, *P* = 0.78). Significant differences in pairwise comparisons (Dunnett's test) were found between WT/vehicle and Thy1-aSyn/vehicle at 8, 12, 16, 20 and 24 weeks of age, that is, after 4, 8, 12, 16, and 20 weeks of treatment, respectively (*P* < 0.01). The decrease in body weight in Thy1-aSyn mice confirms previous findings (Fleming et al. 2004). Body temperatures were affected by genotype (main effect: *P* < 0.001), with a slight decrease in Thy1-aSyn mice, and by age (main effect: *P* < 0.001), but not by drug treatment (Table 3). However, Dunnett's post hoc test did not reveal any significant differences in pairwise comparisons, indicating that the genotype effect is probably due to a minor variability within the groups. Additionally, despite the genotype effect, body temperatures of all experimental groups were within physiological range (37–39°C), indicating that the decrease in temperature observed in Thy1-aSyn mice cannot be considered as hypothermia. Thus, neither dose of NAP had detrimental effects on body weights and temperature.

**Table 2:** Mean (SEM) for body weights (g) recorded during the 24-week drug treatment

Group	4 weeks (treatment initiation)	8 weeks	12 weeks	16 weeks	20 weeks	24 weeks
WT/vehicle (*N* = 22)	22.55 (0.74)	28.04 (0.92)	31.82 (1.17)	36.74 (1.35)	40.13 (1.36)	42.63 (1.46)
WT/2 *μ*g NAP (*N* = 20–21)	22.62 (0.57)	28.33 (0.81)	31.91 (1.00)	36.26 (1.46)	41.55 (1.08)	44.11 (4.43)
WT/15 *μ*g NAP (*N* = 16–17)	22.26 (0.88)	27.16 (1.02)	30.56 (1.20)	35.78 (1.70)	39.16 (2.19)	41.76 (2.38)
Thy1-aSyn/vehicle (*N* = 16–19)	20.41 (0.51)	24.82** (0.66)	27.5** (0.80)	32.06** (1.38)	34.75** (1.57)	35.78** (1.72)
Thy1-aSyn/2 *μ*g NAP (*N* = 16–20)	21.12 (0.38)	25.49 (0.46)	28.93 (0.67)	31.69 (0.75)	32.98 (1.11)	31.69 (0.75)
Thy1-aSyn/15 *μ*g NAP (*N* = 21–25)	21.58 (0.35)	26.06 (0.43)	28.87 (0.52)	31.25 (0.77)	32.90 (0.98)	35.73 (1.11)

*N* denotes number of animals at the end and the beginning of the study, and is variable because of attrition by the end of the study.

** *P* < 0.01 vs. WT/vehicle, ANOVA followed by Dunnett's test.

**Table 3:** Mean (SEM) of body temperatures (°C) recorded during the 24-week drug treatment

Group	4 weeks (treatment initiation)	8 weeks	12 weeks	16 weeks	20 weeks	24 weeks
WT/vehicle (*N* = 22)	38.37 (0.11)	37.87 (0.19)	37.64 (0.24)	37.46 (0.23)	37.33 (0.19)	37.57 (0.3)
WT/2 *μ*g NAP (*N* = 20–21)	38.28 (0.16)	37.79 (0.12)	37.63 (0.22)	37.41 (0.16)	37.00 (0.25)	37.89 (0.03)
WT/15 *μ*g NAP (*N* = 16–17)	38.22 (0.14)	37.74 (0.23)	37.51 (0.31)	37.48 (0.26)	37.27 (0.29)	37.67 (0.34)
Thy1-aSyn/vehicle (*N* = 16–19)	38.53 (0.12)	37.62 (0.17)	37.22 (0.27)	37.08 (0.24)	36.76 (0.19)	37.35 (0.31)
Thy1-aSyn/2 *μ*g NAP (*N* = 16–20)	37.88 (0.18)	37.28 (0.21)	37.04 (0.31)	37.17 (0.21)	37.09 (0.29)	37.01 (0.25)
Thy1-aSyn/15 *μ*g NAP (*N* = 21–25)	38.07 (0.15)	37.56 (0.14)	36.78 (0.3)	37.36 (0.24)	37.01 (0.22)	37.08 (0.19)

There were no significant differences between mice on different treatments (mixed design ANOVA: 2 × 3 × 6, main effect of treatment *F*_2,118_ = 0.68 *P* = 0.5; main effect of genotype *F*_1,118_ = 18.01, *P* < 0.001; main effect of age *F*_5,590_ = 26.11, *P* < 0.001; Bonferroni post hoc test not significant). *N* denotes number of animals at the end and the beginning of the study, and is variable because of attrition by the end of the study.

### Thy1-aSyn mice exhibit brain tau hyperphosphorylation, which is partially decreased by NAP treatment

To assess whether NAP concentrations in brain were sufficient to produce the desired effect of decreasing tau hyperphosphorylation in the transgenic mice, we measured tau phosphorylation as the ratio of p-tau and total tau levels at the end of the experiment in a subgroup of mice from each treatment group. The brain was dissected into cerebellum, cortex, hippocampus, and a subcortical tissue block (“subcortical region”) that included basal forebrain, amygdala, striatum, and SN, all regions containing α-synuclein pathology in PD (Braak et al. 2003). For graphic representation, the mean of WT/vehicle group was set as 100%.

**Figure 1:**
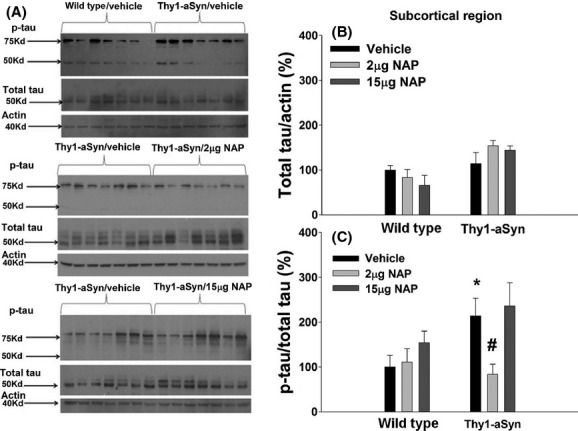
(A) A blot showing actin, total and phosphorylated tau in the subcortical region at 28 weeks of age (after 24 weeks of vehicle or NAP treatment). (B) Densitometry analysis of total tau/actin; *N* = 7 per group. (C) Densitometry analysis of p-tau/total tau; *N* = 7 for all groups except for Thy1-aSyn/2 *μ*g NAP (*N* = 6). Black bars: vehicle; light gray bars: 2 *μ*g NAP; dark gray bars: 15 *μ*g NAP. *P < 0.05 vs. WT/vehicle; ^#^*P* < 0.05 vs. Thy1-aSyn/vehicle, two-way ANOVA followed by post hoc one-tailed Dunnett's test. Data are presented as mean±SEM. Each experiment was replicated three times.

While no significant differences were noted in the total tau level in the subcortical region (Fig. 1A, B), tau phosphorylation levels were significantly modified by main effects of genotype (Fig. 1A, C; two-way ANOVA: *F*_1,35_ = 4.67, *P* < 0.05), treatment (*F*_2,35_ = 3.92, *P* < 0.05) but no interaction (*F*_2,35_ = 2.04, *P* > 0.05). When Dunnett's post hoc analysis was performed, a significant genotype effect was detected, with tau phosphorylation increasing by more than twofold in the vehicle-treated Thy1-aSyn mice (*N* = 7), being 213.7 ± 39.4% of WT/vehicle (*N* = 7) phosphorylation level (*P* < 0.05 vs. WT/vehicle) (Fig. 1C). This indicates that overexpression of α-synuclein increases pathological tau phosphorylation in regions affected in PD even at a young age in these mice. Administration of NAP at 2 *μ*g daily (Monday–Friday) significantly reduced the phosphorylation levels in Thy1-aSyn mice in the subcortical region (*N* = 6, *P* < 0.05 vs. Thy1-aSyn/vehicle, Dunnett's test), where it completely normalized them to 83.6 ± 22.6% of WT/vehicle level. The 15 *μ*g daily NAP dose was ineffective (*N* = 7, 236.2 ± 51.4% of WT/vehicle level; *P* = 0.87 vs. Thy1-aSyn/vehicle) (Fig. 1C).

**Figure 2:**
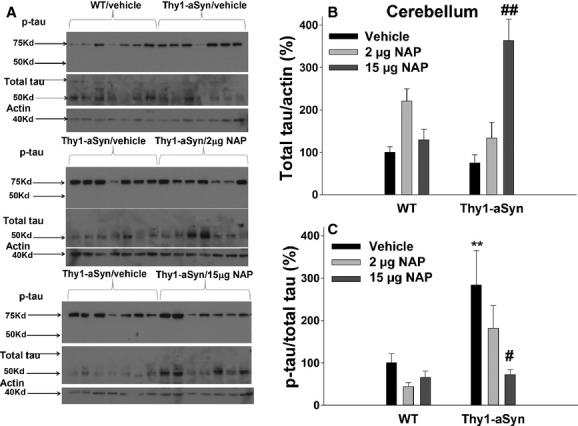
(A) A blot showing actin, total and phosphorylated tau in the cerebellum at 28 weeks of age (after 24 weeks of vehicle or NAP treatment). (B) Densitometric analysis of total tau/actin; *N* = 7 per group. (C) Densitometry analysis of p-tau/total tau; *N* = 7 per group. Black bars: vehicle; light gray bars: 2 *μ*g NAP; dark gray bars: 15 *μ*g NAP. **P < 0.01 vs. WT/vehicle; ^#^*P* < 0.05, ^##^*P* < 0.01 vs. Thy1-aSyn/vehicle, two-way ANOVA followed by post hoc one-tailed Dunnett's test. Data are presented as mean±SEM. Each experiment was replicated three times.

In the cerebellum, like in the subcortical region, there was no genotype effect on total tau levels (Fig. 2A, B, ANOVA: *F*_1,36_ = 2.53, *P* > 0.05). However, there were significant effect of treatment (*F*_2,36_ = 13.01, *P* < 0.01) and an interaction between genotype and treatment (*F*_2,36_ = 14.81, *P* < 0.01). Unexpectedly, an almost fivefold increase in total tau was observed in the Thy1-aSyn mice following the 15 *μ*g NAP treatment (*N* = 7, 363.66 ± 50.2% of WT/vehicle levels vs. 74.8 ± 19.6% of WT/vehicle levels in the vehicle-treated Thy1-aSyn mice [*N* = 7, Dunnett's test: *P* < 0.01]). Phosphorylated tau levels in the cerebellum were modified by significant genotype (*F*_1,36_ = 10.35, *P* < 0.01) and treatment (*F*_2,36_ = 4.45, *P* < 0.05) effects, with no significant interaction effect (*F*_2,36_ = 2.41, *P* > 0.05). An almost threefold increase in phosphorylated tau levels was found in Thy1-aSyn/vehicle mice (*N* = 7; 283.6 ± 81.6% of WT/vehicle) compared to WT/vehicle mice (*N* = 7) (Fig. 2A, C; *P* < 0.01, Dunnett's test). The 2 *μ*g daily NAP treatment did not significantly reduce the phosphorylation levels in Thy1-aSyn mice in the cerebellum compared to Thy1-aSyn/vehicle (Fig. 2C, 181.85 ± 53.2% of WT/vehicle level [*N* = 7] vs. 283.6 ± 81.6% of WT/vehicle, respectively). The 15 *μ*g daily NAP treatment reduced the apparent phosphorylation level in Thy1-aSyn mice, to 72.7 ± 11.1% of WT/vehicle level (*N* = 7, *P* < 0.05 vs. Thy1-aSyn/vehicle), however this reduction in p-tau was driven by the massive increase in T-tau (Fig. 2B; Total Tau/Actin) since the p-tau data is expressed as a ratio of p-tau to T-tau.

Thus, a significant increase in tau phosphorylation was seen in both the subcortical region and the cerebellum of the Thy1-aSyn mice when compared to WT animals. This hyperphosphorylation was reduced to control levels in the subcortical region by the 2 *μ*g NAP, whereas the high dose of NAP (15 *μ*g) resulted in a decrease in tau phosphorylation in the cerebellum but not in the subcortical region. These data indicate that, in our experimental cohort, NAP did reach effective concentrations in brain to produce changes in tau phosphorylation as intended, although, in the cerebellum, the dose-dependent increase in total tau induced by NAP complicates the interpretation of the changes in p-tau. Unfortunately, data from the cortical and hippocampal tissues were highly variable, perhaps due to individual animal variability or tissue dissection and preparation, which precluded further analysis in these brain regions.

### NAP improves behavioral alterations in the open field in young Thy1-aSyn mice

Data of motor activity in the open field were collected 10 days before sacrifice, that is, when mice were 27 weeks old and had been administered NAP for 23 weeks. Previous studies revealed hyperactivity of young (4–7 months of age) Thy1-aSyn mice in the open field (Lam et al. 2011; Wang et al. 2012), an effect observed in several mouse models of synucleinopathies at a young age (Chesselet and Richter 2011). Activity data were collected automatically by the TruScan system, and we analyzed move time and move time per episode, parameters that showed high power to detect drug effects with the smallest number of animals based on our previous studies (Lam et al. 2011; Chesselet et al. 2012). We analyzed the data for the total 15-min session as well as for each one of the three 5-min time blocks.

#### Genotype effect on total move time

**Figure 3:**
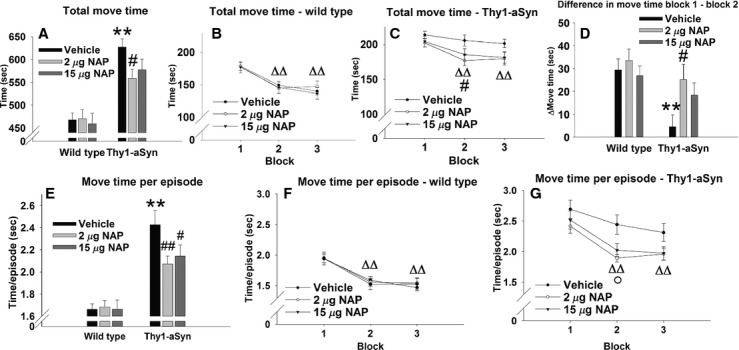
Open field performance at 27 weeks of age (after 23 weeks of treatment). (A–D) Total time spent in motion. (A) Time spent in motion during a 15-min session; *N* = 22 for WT/vehicle; *N* = 20 for WT/2 *μ*g NAP; *N* = 16 for WT/15 *μ*g NAP; *N* = 16 for Thy1-aSyn/vehicle; *N* = 16 for Thy1-aSyn/2 *μ*g NAP; *N* = 22 for Thy1-aSyn/15 *μ*g NAP. Black bars: vehicle; light gray bars: 2 *μ*g NAP; dark gray bars: 15 *μ*g NAP. **P < 0.01 vs. WT/vehicle; #*P* < 0.05 vs. Thy1-aSyn/vehicle, two-way ANOVA followed by post hoc one-tailed Dunnett's test. (B) Total time spent in motion in WT mice, broken into three 5-min bins; vehicle, *N* = 22; 2 *μ*g NAP, *N* = 20; 15 *μ*g NAP, *N* = 16. (C) Total time spent in motion in Thy1-aSyn mice, broken into three 5-min bins; vehicle, *N* = 15; 2 *μ*g NAP, *N* = 16; 15 *μ*g NAP, *N* = 22. Black circles: vehicle; open circles: 2 *μ*g NAP; black triangles: 15 *μ*g NAP. ΔΔ*P* < 0.01 vs. block 1 in all WT groups, and in 2 *μ*g and 15 *μ*g NAP-treated Thy1-aSyn groups, paired *t*-test; #*P* < 0.05, 2 *μ*g NAP vs. vehicle, two-way repeated measure ANOVA followed by post hoc one-tailed Dunnett's test. (D) Difference in move time between block 1 and block 2; *N* = 22 for WT/vehicle; *N* = 20 for WT/2 *μ*g NAP; *N* = 15 for WT/15 *μ*g NAP; *N* = 17 for Thy1-aSyn/vehicle; *N* = 16 for Thy1-aSyn/2 *μ*g NAP; *N* = 21 for Thy1-aSyn/15 *μ*g NAP. Black bars: vehicle; light gray bars: 2 *μ*g NAP; dark gray bars: 15 *μ*g NAP. **P < 0.01 vs. WT/vehicle, #*P* < 0.05 vs. Thy1-aSyn/vehicle, two-way ANOVA followed by post hoc one-tailed Dunnett's test. (E–G) Move time per episode. (E) Move time per episode during a 15-min session; *N* = 22 for WT/vehicle; *N* = 20 for WT/2 *μ*g NAP; *N* = 16 for WT/15 *μ*g NAP; *N* = 17 for Thy1-aSyn/vehicle; *N* = 16 for Thy1-aSyn/2 *μ*g NAP; *N* = 21 for Thy1-aSyn/15 *μ*g NAP. Black bars: vehicle; light gray bars: 2 *μ*g NAP; dark gray bars: 15 *μ*g NAP. **P < 0.01 vs. WT/vehicle; ^#^*P* < 0.05, ^##^*P* < 0.01 vs. Thy1-aSyn/vehicle, two-way ANOVA followed by post hoc one-tailed Dunnett's test. (F) Move time per episode in WT mice, broken into three 5-min bins; vehicle, *N* = 22; 2 *μ*g NAP, *N* = 20; 15 *μ*g NAP, *N* = 16 (G) Move time per episode in Thy1-aSyn mice, broken into three 5-min bins; vehicle, *N* = 21; 2 *μ*g NAP, *N* = 20; 15 *μ*g NAP, *N* = 16. Black circles: vehicle; open circles: 2 *μ*g NAP; black triangles: 15 *μ*g NAP. ΔΔ*P* < 0.01 vs. block 1 in all WT groups, and in 2 *μ*g and 15 *μ*g NAP-treated Thy1-aSyn groups, paired *t*-test; °*P* < 0.05 for 2 *μ*g and 15 *μ*g NAP-treated vs. vehicle-treated mice, repeated measure ANOVA followed by post hoc one-tailed Dunnett's test. A break in the *y*-axis in (A–C) and (E–G) was used to collapse data to focus on the hyperactivity expressed by Thy1-aSyn mice compared to WT. Data are presented as mean ± SEM.

For total move time, a significant main effect was found for genotype (*F*_1,106_ = 54.43, *P* < 0.01), with no main effect for treatment (*F*_2,106_ = 0.85, *P* > 0.05), or interaction effect (*F*_2,106_ = 1.96, *P* > 0.05, Fig. 3A). A 33% increase in move time was observed indicating hyperactivity in vehicle-treated Thy1-aSyn mice compared to vehicle-treated WTs (WT/vehicle: 468.07 ± 14.96 sec, *N* = 22; Thy1-aSyn/vehicle: 627.59 ± 17.74 sec, *N* = 16, post hoc Dunnett's test: *P* < 0.01), and confirming previous data at this age (Lam et al. 2011).

#### Treatment effect on total move time

A significant improvement of this phenotype was found with daily 2 *μ*g NAP, decreasing move time by 11% compared to vehicle-treated Thy1-aSyn mice (558.75 ± 20.11 sec, *N* = 16; one-tailed Dunnett's test: *P* < 0.05 vs. vehicle-treated Thy1-aSyn mice). One outlier was excluded from the Thy1-aSyn/vehicle group after performing Grubb's test for outliers. An apparently similar decrease with 15 *μ*g NAP did not reach statistical significance.

Previous studies showed that in contrast to WT mice that habituate to the open field with time, Thy1-aSyn mice keep a constant level of activity throughout the three blocks suggesting a deficit in learning in a novel environment (Lam et al. 2011). This was confirmed in the current study with a main effect of the time block on the total move time in WT mice (Fig. 3B, F_2,110_ = 58.31, *P* < 0.01) due to a decrease in move time from block 1 to block 2 and from block 1 to block 3 (*P* < 0.01 for all treatments, paired *t*-test), indicating habituation to the novel environment with time. No significant main effect of treatment (*F*_2,55_ = 0.09, *P* > 0.05), or interaction effect (*F*_4,110_ = 0.66, *P* > 0.05) were found in the WT mice. In the Thy1-aSyn mice, a significant main effect on the total move time was found for the blocks (Fig. 3C, F_2,104_ = 15.06, *P* < 0.01), with no main effect for treatment (*F*_2,52_ = 1.32, *P* > 0.05), or interaction effect (*F*_4,104_ = 1.84, *P* > 0.05). However, paired *t*-test revealed that the vehicle-treated mice did not decrease their move time from the first to the second block or to the third block (*P* > 0.05), indicating no habituation to the novel environment. The main effect of block was mainly due to a decrease in move time from the first to the second time block and from the first time block to the third time block in both 2 and 15 *μ*g treated groups (*P* < 0.01), suggesting improvement by drug treatment. Indeed, Dunnett's test revealed a significant decrease of 15% in move time by 2 *μ*g NAP in block 2 (177.03 ± 7.62 sec, *N* = 16 vs. 208.56 ± 8.12 sec, *N* = 16 for vehicle-treated Thy1-aSyn mice, *P* < 0.05), indicating an improvement of the hyperactive phenotype by the drug in Thy1-aSyn mice in this block, in accordance with increasing habituation.

Because both WT mice (regardless of treatment) and NAP-treated Thy1-aSyn mice habituated to the open field from block 1 to block 2, whereas vehicle-treated Thy1-aSyn mice did not, we decided to analyze the difference in the time spent in motion between block 1 and block 2 across genotypes and treatments (Fig. 3D). Two-way ANOVA revealed a significant genotype effect (*F*_1,106_ = 10.66, *P* < 0.01), but not treatment effect (*F*_2,106_ = 2.36, *P* > 0.05) or interaction effect (*F*_2,106_ = 1.55, *P* = 0.05). Post hoc Dunnett's test revealed a significant decrease of 84% (*P* < 0.01) in Thy1-aSyn/vehicle (4.5 ± 5.2 sec, *N* = 17) compared to WT/vehicle (29.4 ± 4.9 sec, *N* = 22), confirming the absence of any decrease in move time in the former group from block 1 to block 2. However, 2 *μ*g NAP improved habituation of the Thy1-aSyn mice to the open field, increasing the difference block 1 – block 2 by over fivefold, to 25.13 ± 6.71 sec (*N* = 16, *P* < 0.05 vs. Thy1-aSyn/vehicle) and completely normalizing it to WT values. NAP (15 *μ*g) did not improve habituation (*N* = 22, *P* > 0.05 vs. Thy1-aSyn/vehicle). Both 2 and 15 *μ*g NAP-treated Thy1-aSyn mice showed a difference in move time comparable to vehicle-treated WT mice (*P* > 0.05, NS), indicating that the drug treatment normalized the performance of the transgenic mice. We can rule out the possibility that vehicle-treated Thy1-aSyn mice did not decrease their move time due to a lower activity in the first block, because their activity in the first block was higher than that of vehicle-treated WTs (Fig. 3B, C).

#### Genotype effect on move time per episode

Move time per episode was also increased in vehicle-treated Thy1-aSyn mice compared to their WT littermates. A significant main effect was found for genotype (*F*_1,106_ = 59.72, *P* < 0.01), but not for treatment (*F*_2,106_ = 1.34, *P* > 0.05) or interaction (*F*_2,106_ = 3.08, *P* = 0.05). A 45% increase in move time per episode was observed in vehicle-treated Thy1-aSyn mice compared to vehicle-treated WTs (Fig 3E; WT/vehicle: 1.66 ± 0.05 sec, *N* = 22; Thy1-aSyn/vehicle: 2.43 ± 0.13 sec, *N* = 17, *P* < 0.01, Dunnett's test), which was attenuated by 10–15% with both 2 *μ*g NAP (2.07 ± 0.07 sec, *P* < 0.01) and 15 *μ*g NAP (2.14 ± 0.1 sec, *P* < 0.05).

#### Treatment effect on move time per episode

Similar to total move time, an improvement was observed on move time per episode. In the WT group, there was a main effect of the time block on move time per episode (Fig. 3F, *F*_2,110_ = 71.75, *P* < 0.01), again due to a decrease in move time from block 1 to block 2 and from block 1 to block 3 (*P* < 0.01 for all treatments). No significant main effect of treatment (*F*_2,55_ = 0.03, *P* > 0.05) or interaction effect (*F*_4,110_ = 0.47, *P* > 0.05) were found. In the Thy1-aSyn mice, a significant main effect on move time per episode was found for the time block (Fig. 3G, *F*_2,104_ = 23.93, *P* < 0.01), but not for the treatment (*F*_2,52_ = 2.01, *P* > 0.05) or interaction (*F*_4,104_ = 1.19, *P* > 0.05). Similar to total move time, there was no decrease in move time per episode from the first to the second time block in the vehicle-treated Thy1-aSyn group (*P* > 0.05) but NAP-treated groups decreased their activity (*P* < 0.01 for both doses) – confirming again a stronger habituation in the drug-treated than in the vehicle-treated Thy1-aSyn mice. Post hoc Dunnett's test revealed significant decrease (20%) in move time per episode in block 2 with both 2 and 15 *μ*g NAP (*P* < 0.05).

Analyzing the difference in move time per episode between blocks 1 and 2 did not reveal any main effects of genotype or treatment, due to the high variability of the data (not shown).

Thus, 23 weeks of treatment of NAP reduced hyperactivity in the open field in Thy1-aSyn mice, more consistently with the low dose. The primary effect was the shortening of the move episodes, which led to a decrease in total move time. In addition, whereas all WT groups significantly decreased their activity from block 1 to block 2, Thy1-aSyn mice treated with vehicle did not show a decrease in activity until the third block; the low dose of NAP completely restored habituation to the open field in the Thy1-aSyn mice, normalizing it to values obtained in WT mice.

Despite clear improvements in the open field after NAP treatment for 23 weeks, there were no effects of NAP on deficits of motor coordination in the Thy1-aSyn mice. A very reliable and early deficit exhibited by Thy1-aSyn mice is an increase in the number of errors per step in the challenging beam test (Fleming et al. 2004; Chesselet et al. 2012). Contrary to previous results with daily administration of NAP (Fleming et al. 2011), no significant improvement was observed after NAP administration for 5 days a week at either dose, either after 8 or 23 weeks of treatment (Table 4).

**Table 4:** Mean (SEM) of errors/step on the challenging beam averaged on five trials 8 and 23 weeks post treatment, and progression of beam deficits from time point 1 to time point 2, averaged on five trials for the entire beam length and for segment 2 only (two-way ANOVA: 2 × 3, main effect of genotype: *P* < 0.001 for all parameters; main effect of treatment: *P* > 0.05 for all parameters, nonsignificant; interaction effect: *P* > 0.05 for all parameters, nonsignificant)

Group	Errors/step averaged on five trials at time point 1 (8-week treatment)	Errors/step averaged on five trials at time point 2 (23-week treatment)	Difference in errors/step time point 2 − time point 1
WT/vehicle (*N* = 22)	0.07 (0.01)	0.1 (0.01)	0.03 (0.03)
WT/2 *μ*g NAP (*N* = 21)	0.08 (0.01)	0.11 (0.01)	0.03 (0.03)
WT/15 *μ*g NAP (*N* = 17)	0.1 (0.01)	0.11 (0.02)	0.01 (0.03)
Thy1-aSyn/vehicle (*N* = 17–19)	0.42 (0.04)**	0.62 (0.05)**	0.24 (0.03)**
Thy1-aSyn/2 *μ*g NAP (*N* = 16–20)	0.41 (0.03)	0.62 (0.05)	0.21 (0.03)
Thy1-aSyn/15 *μ*g NAP (*N* = 22–25)	0.42 (0.03)	0.57 (0.04)	0.15 (0.04)

*N* denotes number of animals at the second and first time points, and is variable because of attrition by the end of the study.

** *P* < 0.01 vs. WT/vehicle, Dunnett's.

Similar to the beam test, the regimen of NAP administration used in this study had little effect on the pole test (Table 5). When the test was performed after 8 weeks of treatment no overall effect was noted on the average of five trials, the highest dose of NAP improved time to turn on the pole in the first two trials only, and to descend on trial two (*P* < 0.05; one-tailed Mann–Whitney *U* test with Bonferroni correction for multiple comparisons). Older Thy1-aSyn mice become unable to perform the test as shown by the analysis of the number of performers at the two time points, and NAP had no effect on this severe deficit.

**Table 5:** Mean (SEM) of time to turn and time to descend on the vertical pole, averaged for five trials and recorded for individual trials, and percent of performers – mice that were able to turn on the pole and descend it within the 30 sec cutoff

	Time point 1 (8-week treatment)					Time point 2 (23-week treatment)	
		
Group	Average time (five trials) to turn (sec)	Time to turn on trial 1 (sec)	Time to turn on trial 2 (sec)	Time to descend on trial 2 (sec)	% performers	Average time (five trials) to turn (sec)	% performers
WT/vehicle (*N* = 22)	6.3 (1.3)	4.7 (1.3)	6.0 (1.5)	4.7 (1.3)	100.0	21.1 (2.2)	55
WT/2 *μ*g NAP (*N* = 21)	6.3 (1.2)	3.1 (0.3)	5.5 (1.8)	6.1 (1.7)	100.0	22.4 (1.9)	60
WT/15 *μ*g NAP (*N* = 17)	8.8 (2.1)	5.4 (1.6)	6.8 (2.2)	6.8 (2.15)	94.1	22.8 (2.5)	43.3
Thy1-aSyn/vehicle (*N* = 17–19)	26.0 (1.4)**	24.5 (2.5)**	26.0 (2.0)**	28.5 (2.5)**	47.4	30.0 (0)**	0
Thy1-aSyn/2 *μ*g NAP (*N* = 16–20)	27.5 (0.8)	26.2 (1.8)	26.4 (2.0)	28.15 (2.04)	45.0	30.0 (0)	0
Thy1-aSyn/15 *μ*g NAP (*N* = 22–25)	21.4 (1.8)	18.9 (2.7)#	21.3 (2.4)#	20.0 (2.7)#	60.0	30.0 (0)	0

*N* denotes number of animals at the second and first time points, and is variable because of attrition by the end of the study.

** *P* < 0.01 vs. WT/vehicle, two-tailed Mann–Whitney *U* test with Bonferroni correction for multiple comparisons;

# *P* < 0.05 vs. Thy1-aSyn/vehicle, one-tailed Mann–Whitney *U* test with Bonferroni correction for multiple comparisons.

### NAP improves nonmotor functions in Thy1-aSyn mice in a test of olfaction

Motor deficits in PD patients are often preceded by a range of nonmotor deficits, including early alterations in olfaction, which can precede by many years the onset of motor symptoms (Haehner et al. 2014). We assessed olfactory function with the buried pellet test because it provides high power to detect drug effects (Chesselet et al. 2012). The test was administered at 28 weeks of age (i.e., after 24 weeks of treatment, 5 days prior to sacrifice) because the test requires food deprivation and does not show progressive worsening (Fleming et al. 2008). We chose to test the mice as close as possible to the cessation of treatment to ensure maximal effect, because previously, 8 week treatment was not sufficient to improve olfaction, when tested 1 month after cessation of treatment (Fleming et al. 2011).

Mice that were uncovering bedding anywhere other than the vicinity of the pellet 10 times or more were excluded from the analysis because these mice were further burying the pellet, making it more difficult to be detected by smell. Based on this criterion, two mice per group were excluded from the WT groups treated with 2 and 15 *μ*g NAP, and three were excluded from the vehicle-treated WT group. One more mouse was excluded from the Thy1-aSyn/2 *μ*g NAP group. Fisher's exact test revealed that there were no differences in the percentage of mice excluded in the different groups (*P* > 0.05). No difference was found in latencies to find a visible pellet test indicating that there was no difference among groups in interest in food and motor function (WT/vehicle: 49.9 ± 16.4 sec, *N* = 22; Thy1-aSyn/vehicle: 83.6 ± 25.7 sec, *N* = 16; Mann–Whitney: *P* = 0.6).

**Figure 4:**
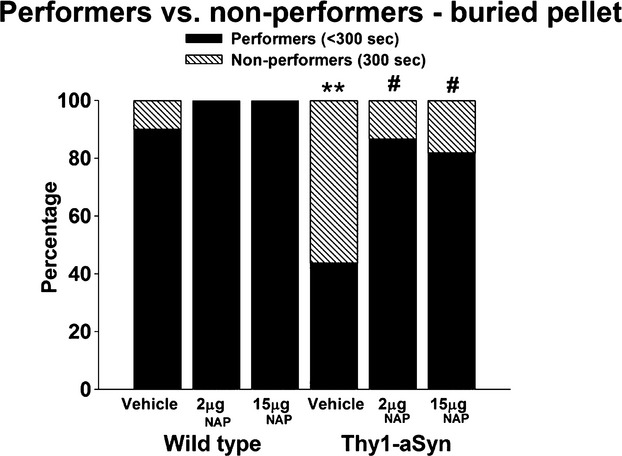
Percentage of performers, mice finding pellet within 300 sec (black portion of bar) vs. nonperformers, mice not finding the pellet (hatched portion of bar) in the olfaction test performed at 28 weeks of age (after 24 weeks of treatment). **P < 0.01 vs. WT/vehicle, ^#^*P* < 0.05 vs. Thy1-aSyn/vehicle, two-tailed Fisher's exact test with Bonferroni correction for multiple comparisons. *N* = 19 for WT/vehicle; *N* = 18 for WT/2 *μ*g NAP; *N* = 16 for WT/15 *μ*g NAP; *N* = 16 for Thy1-aSyn/vehicle; *N* = 15 for Thy1-aSyn/2 *μ*g NAP; *N* = 22 for Thy1-aSyn/15 *μ*g NAP.

Because many Thy1-aSyn mice treated with vehicle reached the threshold for latency to find the buried pellet and were assigned the threshold value, looking at the latency alone might not be informative. Therefore, we examined the data by distinguishing “performers,” that is, mice that find the pellet before the cutoff time of 300 sec and “nonperformers,” that is, mice that reach the cutoff time without finding the pellet. There was a 50% decrease in the percentage of performers in Thy1-aSyn/vehicle group (7/16, 43.25%), compared to WT/vehicle (17/19, 89.5%) (Fig. 4, *P* = 0.027, Fisher's exact test with Bonferroni correction for multiple comparisons). 2 and 15 *μ*g NAP increased the percentage of Thy1-aSyn mice performers to that of WT/vehicle, 86.7% (13/15) and 81.8% (18/22), respectively (*P* = 0.023 and 0.04, respectively, vs. vehicle-treated Thy1-aSyn mice, Fisher's exact test with Bonferroni correction for multiple comparisons). These data indicated that 24 weeks of NAP treatment significantly increased the number of performers in the buried pellet at both doses.

### High-dose NAP increases the size of proteinase K-resistant α-synuclein aggregates in the ventromedial SN

**Figure 5:**
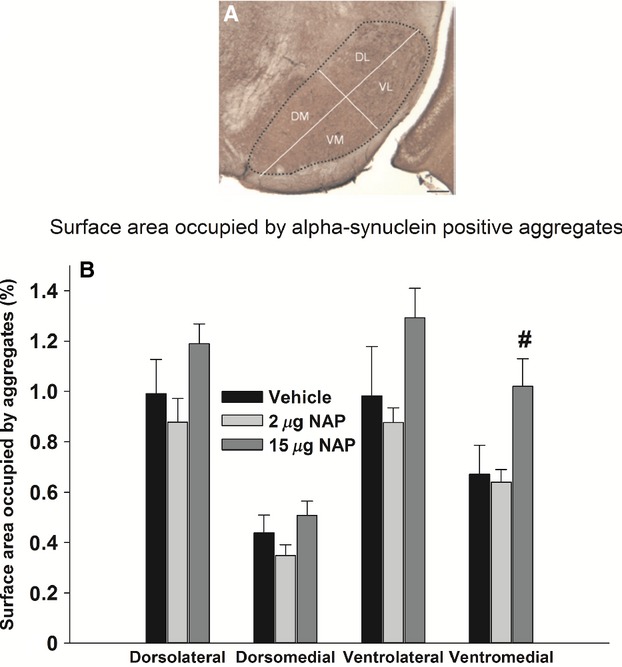
Effect of NAP on proteinase K-resistant α-synuclein aggregates in four subregions of the substantia nigra at 28 weeks of age (after 24 weeks of treatment). (A) Microscopic picture of a coronal section: the dotted line encloses the SN separated into four subregions (scale bar 100 *μ*m). (B) Percentage of surface area occupied by proteinase K-resistant α-synuclein aggregates in the dorsolateral, dorsomedial, ventrolateral, and ventromedial SN of Thy1-aSyn mice. Vehicle, *N* = 8; 2 *μ*g NAP, *N* = 7; 15 *μ*g NAP, *N* = 10. Black bars: vehicle; light gray bars: 2 *μ*g NAP; dark gray bars: 15 *μ*g NAP. ^#^*P* < 0.05 vs. vehicle, one-way ANOVA followed by post hoc two-tailed Dunnett's test. Technical replication was achieved by using four sections per mouse for analysis.

Proteinase K-resistant α-synuclein aggregates are observed in many brain regions of Thy1-aSyn mice, including the olfactory bulb and SN. Because the distribution of aggregates is highly heterogeneous in the SN, aggregates were analyzed separately in the dorsal region, corresponding mainly to the pars compacta and in the ventral region that corresponds to the SN pars reticulata, a region that contains a dense network of dendrites of dopaminergic nigrostriatal neurons as well as sparse efferent GABA-ergic neurons. This regional analysis revealed a significant effect of the high NAP dose on aggregate size in the ventral part of the SN. Significant main effects were revealed on the surface area occupied by aggregates, for both treatment (*F*_2,97_ = 10.248, *P* < 0.001) and subregion (*F*_3,96_ = 21.95, *P* < 0.001). There was more than 30% increase in the surface area occupied by aggregates with 15 *μ*g NAP (*N* = 10) compared to vehicle (*N* = 8) in the ventromedial SN (Fig. 5B, 1.02 ± 0.1% vs. 0.67 ± 0.1%), which was significant (Dunnett's test: *P* = 0.042). No effect was found for 2 *μ*g NAP compared to vehicle (*P* > 0.05, NS). α-synuclein pathology in the olfactory bulb was assessed qualitatively at 20× magnification using a grading scale from 0 to 2 and was not affected by NAP (*P* > 0.05, NS, data not shown), as previously reported (Fleming et al. 2011).

### Microglial activation: IBA-1+ cell density and size distribution

**Figure 6:**
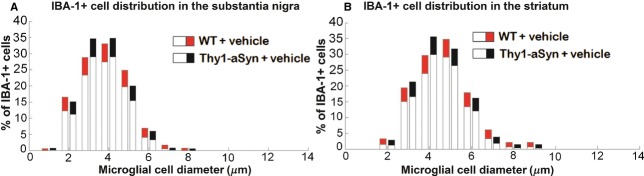
Distribution analysis (bootstrapping) of IBA-1-positive cell diameter in Thy1-aSyn mice treated with vehicle compared to WT mice treated with vehicle at 28 weeks of age (after 24 weeks of treatment) in (A) substantia nigra and (B) striatum. Red and black portions of bar represent the confidence interval of the percentage of IBA-1-positive cells at a certain diameter in WT/vehicle and Thy1-aSyn/vehicle mice, respectively; thus, nonoverlapping black and red portions represent significant difference. No differences were found with bootstrapping. WT/vehicle, *N* = 10; Thy1-aSyn/vehicle, *N* = 8. Technical replication was achieved by using two sections per mouse for analysis.

We have previously observed a significant increase in the diameter of IBA-positive microglial cells in the SN and striatum in Thy1-aSyn mice, indicative of microglial activation (Watson et al. 2012). The red and black portions in the bar histograms represent confidence intervals for each bar in the WT and the Thy1-aSyn group, respectively; thus, nonoverlapping intervals indicate a significant difference. Unexpectedly, no microglial activation was detected in Thy1-aSyn mice administered vehicle compared to vehicle-treated WT mice, as reflected by similar distribution of cells with different diameters in the SN and the striatum, respectively; (Fig. 6A, B). Thus, the genotype effect on microglial activation was not observed in the present study. One possible explanation is that this may have resulted from the use of a vehicle containing benzalkonium chloride, an antimicrobial preservative agent, which could have decreased inflammation in the Thy1-aSyn mice (Jaramillo et al. 2012). NAP administration did not cause any change in microglia diameters compared to mice of the same genotype administered vehicle only. As previously reported, there was no difference in the density of IBA-positive cells in the SN between WT and Thy1-aSyn mice, with or without treatment (two-way ANOVA: main genotype effect: *F*_1,53_ = 1.285, *P* = 0.262, main treatment effect: *F*_2,52_ = 0.619, *P* = 0.543, interaction effect: *F*_2,52_ = 0.78, *P* = 0.46; WT/vehicle: 3.88 ± 0.32 cells/10^4^ *μ*m^2^, *N* = 10; Thy1-aSyn/vehicle: 3.95 ± 0.23 cells/10^4^ *μ*m^2^, *N* = 8). Differences in the striatum were not found either (two-way ANOVA: main genotype effect: *F*_1,51_ = 1.687, *P* = 0.2, main drug effect: *F*_2,50_ = 1.149, *P* = 0.326, interaction effect: *F*_2,50_ = 0.482, *P* = 0.621; WT/vehicle: 0.96 ± 0.06 cells/10^4^ *μ*m^2^, *N* = 10; Thy1-aSyn/vehicle: 1.07 ± 0.04 cells/10^4^ *μ*m^2^, *N* = 8).

## Discussion

The neuroprotective peptide NAP, a drug candidate, has been clinically evaluated under the generic name of davunetide. We have examined the effects of NAP in a model of synucleinopathy at an early disease stage. Thy1-aSyn mice reproduce many aspects of PD, including a striatal dopamine loss with l-dopa-responsive motor deficits at 14 months of age, proteinase K-resistant α-synuclein aggregates, inflammation, mitochondrial dysfunction, and progressive motor and nonmotor deficits (Chesselet et al. 2012). A major advantage of this model is the presence of numerous endpoint measures with high power to detect drug effects at an early age. This is conducive to cost-effective preclinical drug trials for evaluation of potential novel targets for neuroprotection.

Previously described motor and olfactory deficits (Fleming et al. 2004, 2006, 2008, 2011) and hyperactivity (Lam et al. 2011; Wang et al. 2012) in the Thy1-aSyn mice were observed in the present study, confirming the reproducibility of these phenotypes as robust and reliable measures of α-synuclein-induced neuronal dysfunction. We have previously observed beneficial effects of a short (8 weeks) daily NAP administration in this model (Fleming et al. 2011). However, the previous pilot trial only included one dose and no biochemical measurements of target effects were included. The present study was motivated by the need to confirm those preliminary results with a longer administration time, two doses of the compound, and additional endpoints.

We first wanted to confirm target engagement by NAP in our experimental conditions by measuring its effect on tau phosphorylation in brain. Hyperphosphorylation of tau was previously shown in genetic mouse models overexpressing mutant human α-synuclein (Wills et al. 2011) or models expressing WT α-synuclein predominantly in the cortex (Haggerty et al. 2011; Kaul et al. 2011) which reproduce Lewy body disease more closely than PD. Our findings extend these observations to subcortical regions and cerebellum, regions which show numerous proteinase K-resistant aggregates in Thy1-aSyn mice (Zhu et al. 2011). This suggests similarity between the regional patterns of α-synuclein aggregation and tau hyperphosphorylation. Indeed, previous studies in toxin-induced animal and cellular PD models show that α-synuclein pathology contributes to tau aggregation (Duka et al. 2006, 2009). α-synuclein binds to tau and stimulates the protein kinase A-catalyzed tau phosphorylation of serine residues 262 and 356 (Jensen et al. 1999). Our studies verified these in vitro observations, with increased phosphorylation of the serine residue 262 of tau in the subcortical region and cerebellum. Together, these data suggest a potential synergism between α-synuclein and tau pathologies.

In the present study, the effect of two doses (2 and 15 *μ*g/mouse per day) of NAP administered 5 days a week for 24 weeks were evaluated. The dose of 15 *μ*g/mouse per day induced a marked decrease of tau hyperphosphorylation in cerebellum, while the dose of 2 *μ*g/mouse per day decreased p-tau in subcortical regions, including the SN. Neither dose affected tau levels in the subcortical tissue, but NAP 15 *μ*g/mouse per day increased tau in cerebellum, indicating regional differences in the mechanisms underlying the effects of NAP on p-tau/tau ratio. Thus, NAP does affect tau hyperphosphorylation in brain in our experimental conditions, but only the lower dose decreases p-tau in the tissue block containing the region most affected in PD, suggesting a narrow therapeutic margin. This phenomenon of bell-shaped curve was observed before for NAP (Leker et al. 2002; Jouroukhin et al. 2013).

Although the effects of NAP on tau phosphorylation indicate that the drug did reach a sufficient brain level to exert the desired effect, it is unclear that they are mechanistically linked to the behavioral improvements observed after NAP administration. Indeed, the variations in NAP effects on tau phosphorylation were not reflected in their effects on behavioral deficits in the Thy1-aSyn mice; both doses inhibited hyperactivity in the open field and protected against olfactory deficits in Thy1-aSyn mice, even though the effects of the higher dose were attenuated compared to those of the lower dose. A link between tau hyperphosphorylation and hyperactivity was reported in mice expressing mutant human tau (N279K), which presented tau hyperphosphorylation and hyperactivity (Taniguchi et al. 2005). However, 2 *μ*g NAP did not completely normalize hyperactivity despite full normalization of tau hyperphosphorylation, suggesting contribution of other mechanisms to hyperactivity. Because NAP reduces hyperactivity and olfactory deficits without affecting aggregate surface area in the SN, both of these effects could be mediated via mechanisms downstream to α-synuclein aggregation. For instance, NAP could exert its effect by enhancing tau–microtubule interactions, which would prevent free tau accumulation and its potential contribution to its phosphorylation (Oz et al. 2012; Quraishe et al. 2013). In neuronal-like cells with an inherent mitochondrial impairment derived from PD patients, an in vitro PD model, NAP improved microtubule-dependent trafficking, restored the autophagic flux, and potentiated autophagosome–lysosome fusion leading to autophagic vacuole clearance (Esteves et al. 2014). Moreover, NAP was capable of efficiently reducing α-synuclein oligomer content and its sequestration by the mitochondria. Most interestingly, NAP decreased mitochondrial ubiquitination levels as well as increased mitochondrial membrane potential indicating a rescue of mitochondrial function (Esteves et al. 2014). Additionally, NAP increased the interaction of its parent protein, ADNP with microtubule-associated protein 1 light chain 3 (LC3), a key player in the autophagy pathway (Merenlender-Wagner et al. 2013). It is unclear whether NAP produces similar effects in vivo and the design of this study, which was focused on behavioral assessment and tau phosphorylation from the onset, did not permit additional measurements of these newly identified parameters that are improved by NAP in vitro. It is possible that the lack of clear effect of NAP on deficits of motor coordination, including some of the deficits that were ameliorated by a shorter treatment in a previous experiment (Fleming et al. 2011), results from a lack of effect on these pathological endpoints, and future dose-finding experiments based on these novel findings will be necessary to further evaluate NAP potential for the treatment of PD.

One main difference between the present study and our previous experiments that detected beneficial effects of NAP on the beam after 8 weeks of treatment was the schedule of administration. In the present study, NAP was administered Monday to Friday as opposed to our pilot study where NAP was administered daily. The intermittent regimen was chosen for long-term drug administration because it was previously shown to be beneficial in a mouse model of AD and frontotemporal dementia (Matsuoka et al. 2007, 2008; Vulih-Shultzman et al. 2007; Shiryaev et al. 2009; Jouroukhin et al. 2012, 2013). Our current results suggest that sustained NAP exposure to the brain may be required to oppose the severe deficits in motor coordination induced by α-synuclein overexpression. Notably, the deficits on beam and pole performance are established in the Thy1-aSyn mice many months before the appearance of hyperactivity, and were already present when the treatment was initiated (Fleming et al. 2004). Thus, the regimen used in this study was able to prevent the development of additional deficits in the open field, which may include a learning component (lack of habituation to a novel environment) but not reverse established deficits in motor coordination. The importance of repeated NAP exposure for behavioral improvement is also illustrated by the novel observation of significant improvement of olfactory deficits, whereas these deficits were not improved by NAP when measured 1 month after cessation of treatment in our previous study (Fleming et al. 2011). Similarly, in the previous study 2 *μ*g NAP reduced α-synuclein aggregation in the SN after 8 weeks of treatment and a following washout period, but did not have an effect in the present study, despite longer duration of treatment not followed by washout; this is probably due to a progression of α-synuclein pathology with time, in such a way that NAP could no longer prevent it even with a longer administration. Indeed, the surface area occupied by aggregates assessed in the present study at 28 weeks was higher than that assessed in the previous study at 4.5 months (Fleming et al. 2011). Considering the outcome of both studies, it appears that NAP treatment needs to be performed daily in order to maximize the potential benefit. This is consistent with the known, short plasma half-life (∼1 h) of NAP (Morimoto et al. 2009).

In conclusion, the present study demonstrates that the small neuroprotective peptide NAP, when administered intranasally to mice overexpressing α-synuclein, decreases tau hyperphosphorylation and improves a subset of motor and nonmotor deficits exhibited by these mice at a young age, although a direct correlation between the biochemical and behavioral effects could not be established. Both doses were well tolerated and had no adverse effects. Together with recent findings of beneficial effects of NAP in a cell model of PD and in view of our previous data with continuous drug administration, these results support the hypothesis that microtubule stabilizing agents should be further examined for their potential benefit in the treatment of PD. The results of this study provide valuable information for the design of these future preclinical trials.

## Abbreviations

LC3: light chain 3
RM: repeated measure
ANOVA: analysis of variance
HRP: horseradish peroxidase
PCR: polymerase chain reaction
UCLA: University of California Los Angeles
ADNP: activity-dependent neuroprotective protein
MAPT: microtubule-associated protein tau
DAB: diamino benzidine
DL: dorsolateral
DM: dorsomedial
IBA-1: ionized calcium binding adaptor molecule 1
MATLAB: Matrix Laboratory
NAP: the eight-amino acid peptide NAPVSIPQ
PBS: phosphate-buffered saline
SN(c): substantia nigra (pars compacta)
Thy1-aSyn: mice overexpressing α-synuclein under the Thy1 promoter
VL: ventrolateral
VM: ventromedial
WT: wild type
